# Improving Access to Safe Water in Rural Schools of Kenya: Qualitative Multisectoral Insights

**DOI:** 10.7759/cureus.49174

**Published:** 2023-11-21

**Authors:** Peter Kirira, Fiona Oyatsi, Ashley Waudo, Samuel Mbugua

**Affiliations:** 1 Pharmaceutical Chemistry, Mount Kenya University, Thika, KEN; 2 Biochemistry, Strathmore University, Nairobi, KEN; 3 Water Backpack Program, Partners for Care, Nairobi, KEN; 4 Community Health Nursing, Mount Kenya University, Thika, KEN

**Keywords:** social capital, hygiene and sanitation, school-going children, multisectoral collaboration, safe water

## Abstract

Background: Comprehensive strategies in water, sanitation, and appropriate hygiene behaviors can improve school enrolment and improve gender parity disparities. Lack of safe drinking water negatively impacts the social capital of people, especially school-going children in rural areas. In this study, we systematically evaluated and documented evidence on the barriers and facilitators in the access and adoption of safe water practices in rural schools in Laikipia County, Kenya.

Methods: An ethnographic formative, collaborative implementation research design was used in an iterative and participatory process to evaluate community, socio-economic, and health system-related factors affecting water, hygiene, and sanitation strategies. Qualitative data was collected using key informant interviews (n=5) and focus groups (n=3) from various multisectoral participants. Directed content analysis was used to develop codes, categories, and themes from textual data. Data was organized according to the Promoting Action on Research Implementation in Health Services framework.

Results: The findings were classified and described under three key elements: context, evidence, and facilitation. Contextual elements showed an association of diarrhea outbreaks with unsafe hygiene practices compounded by water scarcity. The evidence elements were indicative of the applicability of water backpacks in strengthening handwashing, storage, and transport of water. Facilitation elements indicated evidence of gaps in synergy between the school health and public health systems, necessitating multisectoral collaboration and social capital capacity building.

Conclusion: The national and county governments play an imperative role in ensuring access and continuous supply of safe drinking water in schools. This is fundamental in efforts towards reducing social inequalities of health among school-going children and building their social capital. Participatory, collaborative, multisectoral interventions and decision-making are crucial, leveraging on creating local ownership, in meeting the water consumption needs of children and communities in water-scarce regions.

## Introduction

Diarrhea accounts for 297,000 deaths in children under five years and 929,000 deaths in all population groups globally resulting from unsafe drinking water, sanitation, and hand hygiene (WASH) [1]. This translates to more than 1,000 deaths in under five-year-olds daily from WASH-related diseases [2]. The use of unsafe drinking water sources is a substantial contributor to diarrheal diseases [3]. Sustainable Development Goal 6, target 6.1. calls for universal and equitable access to safe and affordable drinking water for all. Safe drinking water, sanitation and hygiene, and appropriate hygiene behaviors, collectively referred to as WASH, can reduce exposure to enteric micro-organisms [4]. Waterborne diseases are considered an essential indicator of health in deprived populations [5,6], with an estimated 45.8 per 100,000 deaths in Africa attributed to WASH diseases.

Numerous school-going pupils in developing countries miss school or have ineffective schooling due to diseases associated with unsafe drinking water and inadequate sanitation [7]. Approximately 400 million children in developing countries, an average of one in every five children, have no access to safe water. This is exacerbated by the fact that four out of five children use surface water or walk for more than 15 minutes to find a protected water source [8]. Basic education outcomes will be difficult to attain and sustain without safe water in schools, for drinking, handwashing, food preparation, and general hygiene and sanitation [9]. In Kenya, two in five Kenyans are aged between four years and 17 years [10].

The importance of point-of-use water treatment with chlorine as a low-cost water treatment option as a component of WASH strategies is well documented in the literature [11,12]. However, substantial barriers resulting in low utilization of WaterGuard (Population Services International, Washington, D.C., United States), comprised of dilute chlorine, in households have been reported [13], indicative of the vital desideratum of behavioral change communication.

Increased school enrolment and improved disparities in gender parity can be achieved using a comprehensive WASH program that incorporates improved water sources [14]. Safely managed water in schools greatly contributes to the Kenyan government’s goal of providing quality education and training in line with targets set to achieve Vision 2030, and a necessity to several provisions outlined in the constitution [9]. In a study evaluating the impact of a safe school-based water and hygiene program in rural schools in Western Kenya, O’Reilly et al. indicated that the program resulted in a 35% reduction in absenteeism [15]. Improved access is a critical elemental component in ensuring the combating of diarrheal diseases, promoting hygiene, improved school attendance, productive time utilization, and women empowerment [3]. There is compelling evidence of the positive influence of school water and hygiene on educational outcomes. The promotion of hygiene and a water treatment intervention resulted in a significant reduction in school absenteeism by 58% among girls [16]. In Laikipia County’s sub-basin, the main source of water is rivers [17]. Schools lack basic water services with students having to fetch water from the river and/or boreholes. This can result in missing classes and waterborne diseases in this vulnerable population [18].

The scarcity of safe drinking water negatively impacts the social capital of people, especially school-going children, living in rural areas of Kenya. Social capital, referring to features of social organization that facilitate cooperation and coordination for mutual benefit [19,20], is a pivotal component of community-based interventions and has been associated with better social well-being and community resilience. Our research team previously demonstrated the benefits of enhancing safe water in supporting nutritional interventions [21]. In this study, we systematically evaluated and documented evidence on the barriers and facilitators in the access and adoption of safe water practices in rural schools in Laikipia County. This qualitative study provides reflections of various stakeholders in schools, community, and governance on safe water access and adoption. 

This manuscript was previously posted to the Research Square preprint server on July 18, 2023.

## Materials and methods

**Figure 1:**
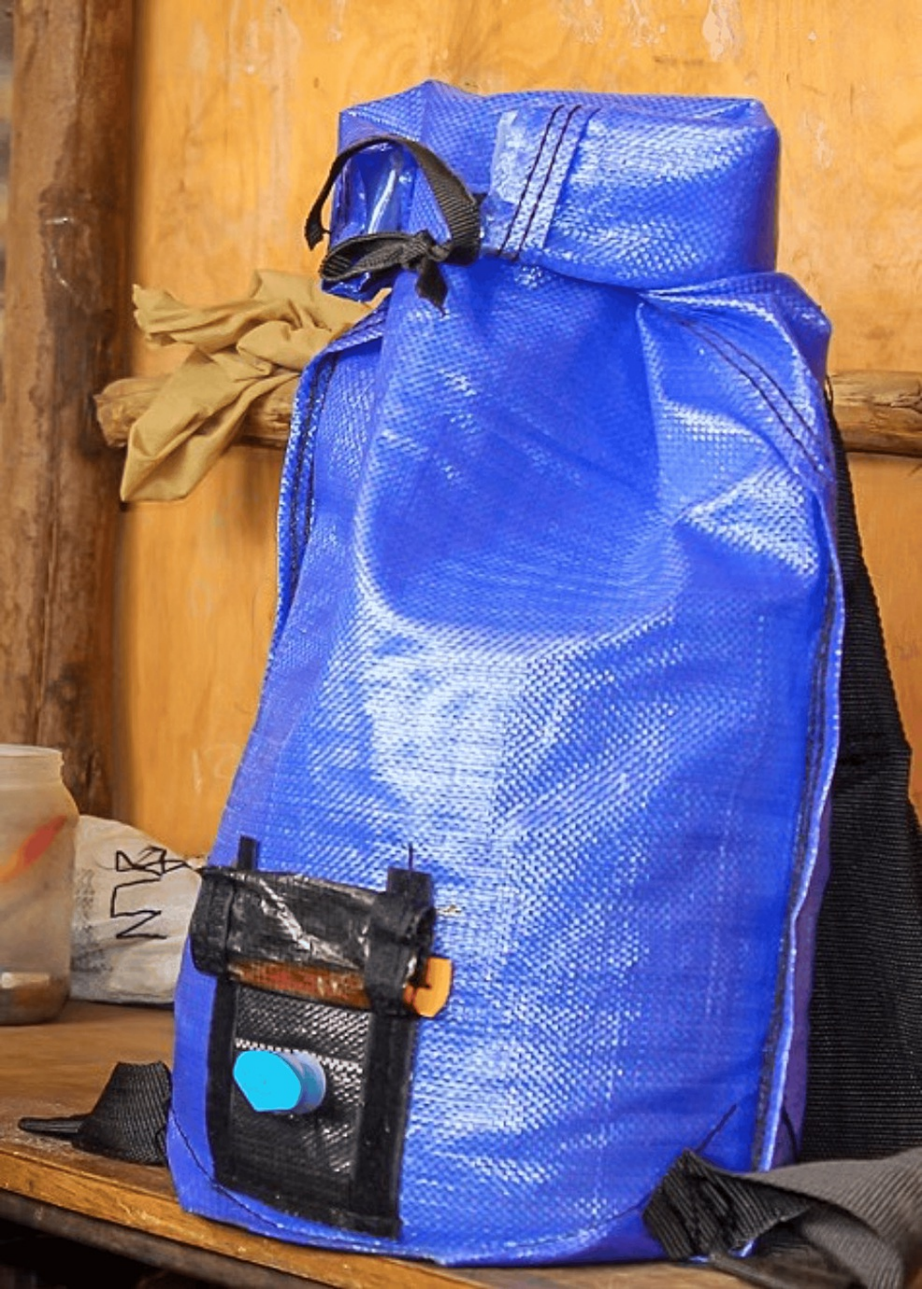
The water backpack Image Courtesy: Partners for Care, Kenya

The study was conducted in Laikipia County, Kenya. Participants were purposively selected from various multisectoral, multilevel levels of governance and programmatic service delivery relevant to WASH strategies. The study evaluated the lived experience, daily routines, and expected change in behavior using school-based and community-centric approaches, to assess the integration process of the intervention in the form of the innovative water backpack (PackH2O) (Figure 1).

PackH2O is an innovative product designed to provide a cleaner, safer alternative to the often-contaminated jerry cans used by children and women in developing countries for water transport, storage, and dispensing. Greif PackH2O Water Backpack (Greif, Inc., Delaware, Ohio, United States) sold the patent to Partners for Care (PFC; Fulton County, United States). In Kenya, PFC is the only producer of water backpacks; however, PFC may take the help of companies for distribution as part of corporate social responsibility programs. The water backpack offers the most benefit in the school set-up as one water backpack impacts 30-40 children unlike at the household level where one water backpack will only impact approximately five individuals in the home. In 2021, 1000 water backpacks were installed to be used as safe water dispensers in 66 resource-constrained public primary schools in Laikipia County. The backpacks were distributed to each class and training was conducted on staff and pupils on their appropriate use.

Research design and data collection

An ethnographic formative, collaborative implementation research design was adapted using an iterative and participatory process to evaluate community, socio-economic, and health system-related factors affecting water, hygiene, and sanitation strategies. A three-pronged approach was used to ensure data saturation and reflexivity: a detailed research log of participants, field notes to account for perspectives and observations, and a research journal with the researcher's thoughts and questions. A secondary aim of the study was to assess the fidelity of the utilization of water backpacks by pupils as an innovative resource aiding in water transportation, storage, and dispensing.

Five key informant interviews (KIIs) and three focus group discussions (FCDs) were conducted. The focus groups comprised headteachers, deputy headteachers, and teachers selected from 20 schools among the 66 that received the water backpacks. The participants were recruited during April 3-12, 2023. The views and opinions of the teachers are fundamental to understanding the pupils', school’s, and the community’s barriers and facilitators to safe water. The key informants were purposively selected and comprised the public health officer (PHO), early childhood development officer (ECD), two senior community health volunteers (CHVs), and the local Member of the County Assembly (MCA). The PHO provided insights on the role of the healthcare system in school-based and community-centric WASH strategies, the education officer provided perspectives on the role of the Ministry of Education in the overseeing of school-based interventions, the CHVs provided community-centric perspectives on the synergy between school health and community health, and the MCA was instrumental in alluding to the role of governance structures in ensuring safe water for schools and communities [22]. The MCA is the political representative in charge of an administrative ward within the devolved governance structures of the Republic of Kenya. 

Data analysis

Qualitative data was collected using audio tapes and field notes, translated, transcribed, and exported into Nvivo v12 (QSR International, Burlington, Massachusetts, United States) for analysis. Directed content analysis processes were used to develop codes, categories, and themes from textual data. Data was organized according to the Promoting Action on Research Implementation in Health Services (PARIHS) framework that describes the successful implementation of the intervention as a function of nature and quality of evidence, characteristics of the context, and the facilitation strategies. This was employed as a two-stage process. Experiences and perceptions on the utilization of water backpacks were evaluated using qualitative techniques to generate narratives. The context elements including the culture, community leadership, and receptivity of the intervention were also assessed qualitatively. Evidence elements entailed the needs and preferences of school-going children and the community and the local practice of ensuring safe drinking water in the schools.

The analysis of qualitative narratives involved transcribing the interviews verbatim and transcription. The second iterative reading refined the emergent themes. The final analysis contained context, evidence, and facilitation elements relevant to the successful implementation of water backpacks in WASH strategies.

Ethical considerations

Ethical clearance was sought from Mount Kenya University’s Institutional Scientific Ethics Review Committee (approval number: MKU/ISERC/2642). All research activities and data handling were in strict compliance with national and global ethical standards. Written informed consent was obtained from each participant before the conduct of an interview. Personal identifiers were not included during data collection, entry, and analysis.

## Results

WASH strategies in school health programs

The participants suggested that pupils in water-constrained schools were required to each bring water for daily utilization from home or "protected water sources". The participants strongly associated the outbreak of diarrhea in schools with unsafe water and poor hygiene practices among pupils.

“…… you'll tell pupils to come with water tomorrow and maybe that water is from cooking their own food, you'll find that some of the boys spit in that water, others add urine (laughter) to that water. And this we just get report from other pupils’ teacher, such as so and so added urine or saliva in that water so as we say, they think that they are being punished. But you see that water is theirs to use in school.” (FGD, Teacher)

“or that water you've used in handwashing, you'll see the kids washing their face with it. When they are washing their face with it, there can be this bacterium, like this one we were saying the kids were having diarrhea.” (KII, Senior CHV)

In some instances, the poor or insufficient hygiene practices stem from the child’s household, indicative of salient risks to the health of the child and the family. There was an appreciation of the water packs as an integral intervention in the pathway of ensuring water safety.

“We have containers at home for the herbs and other chemicals that are used in the shambas (plantations). You find that pupils, because they don't have good containers, they just take those containers and they put water, so when you are smelling that water, you find that they are for the herbicides, so thank you for the waterpacks because we teachers, we make sure that the water that is being put in those containers they are very clean for all of us, even our staff we have one; we just drink water from this class….” (FGD, Teacher)

There were conflicting perspectives on the health system’s synergistic interaction between the school health and public health systems. Some respondents, as indicated by these narratives from the MCA and CHV, opined of evident school health monitoring and pupil education on hygiene and sanitation.

“And we go and check how those children sleep, the way they are treated there in the dormitories by the matron. So, we teach them cleanliness mostly inside the dormitories and outside and if they are girls, the way they should be kept.” (KII, Senior CHV)

“In each area we have public health officers who are supposed to be visiting each school facility now and then and ensure that health standard is up to date. And that is how we follow up. And in case there is any challenge, you know, we also interact with the parents in case they realize they is something that is not adding up well, they just let us know and by then we make a follow up. Yeah, so if the officer in that area hasn’t been able to identify it, they make that, after the follow up, they make that effort of pushing and now the officer making a follow up to ensure that all the requirements are really upheld.” (KII, MCA)

Availability of safe drinking water in schools and communities

There is a perennial lack of/shortage of safe drinking water resulting from adverse environmental conditions.

“those areas.. especially where I am from, there's no water. That's one thing that gives us a problem.” (KII, Senior CHV)

This scarcity of water results in children and community members having to travel long distances to fetch water, which is sometimes not safe for consumption.

“I saw where others go to fetch water is far, it's about a kilometer. Especially when there was drought, some were using money for water to be brought to them, that one is now a challenge. We don't have water; if we had water we could be using it." (KII, Senior CHV)

Local government provided water utility services in some areas but generally, communities and schools resorted to surface water (springs) and digging wells to meet their water needs.

“… we’ve got proper water supply from the water company to some of the schools. But in some areas, they don’t have such supply. Those are the areas that we have had challenges.” (KII, MCA)

“There is a lot of water shortage; in fact, most of our schools do not have piped water and you find that the pupils go to the nearby well or maybe there is a spring somewhere so they go and fetch water using the water bags and they come and hang them in the school.” (KII, ECD)

In some areas, schools must purchase water to meet their sustenance needs during the drought season.

“Like the time we had the drought; the water flow within the school was very limited and so we used to buy water from outside. After the water is brought to the school, it is distributed in those bags so every class has its own water.” (FGD, Teachers)

Synergy between school and public health systems

Some participants from the focus groups felt there was a disconnect between the public health department and schools with one respondent saying that health workers only visit schools during outbreaks, deworming, or immunization. This is suggestive of a need for multisectoral collaboration in health and social capital policy generation.

“In my school, I think they don't come regularly, they take some time. Maybe once a term.” (FDG, Teacher)

“They have not, they have not! Because the time you see them coming in is when there is an outbreak, maybe they're immunizing or they are giving this..this tablet for deworming. That time you will see them in schools.” (KII, ECD)

Some of the roles of the public health department in school health that were mentioned included identification of health challenges in schools, finding solutions to these challenges, routine inspections, ensuring compliance with public health safety codes and standards, and advocacy on handwashing.

"Each term, I am supposed to visit each school and identify the health challenges that are in schools, liase with the administration on the ways to resolve the health issues encountered in the schools. And then we also give notices on how to effect those changes when they are not complying. So we may write a notice to the institution, especially when you give verbal notice once or twice and it is not responded to. And water is very crucial when it comes to health of the children in schools. We also advocate on handwashing facilities in schools.” (KII, PHO)

“The public health officers do come to schools for routine inspections, inspections in the toilets and classrooms, yes inspections.” (FGD, Teacher)

Role of CHVs

The respondents acknowledged the CHVs as an essential facet of community needs assessment and sensitization.

“…..in our line of duty, we usually liaise with CHVs who are the members of health service delivery. CHVs are in touch with the members of the community because they are the ones used to identify challenges and health problems in the community. So even with respect to the water backpacks, they are the ones who can disseminate that information to the community.” (KII, PHO).

The CHVs were perceived as critical community resource persons with a vital role in health promotion and prevention of disease.

“In the same point, I also felt like, in our communities, there are people called voluntary health workers. They know each household in their area. So, they understand the situation in various homes, those families that are not able to treat their water, where they get their water from, etc. They go around and sensitize members of the community the way they sensitize regarding vaccination and using the toilet. I think the community has embraced them, they understand them, they come to tell us about hygiene and so if we involve them they can deliver the message to the community.“ (FGD, Teacher).

Community and WASH

The participants reflected on the role of the community in ensuring the availability of safe and hygienic water. Clean and hygienic water and practices result in higher class attendance, reduction in mortality and morbidity, and higher retention rates in schools.

“You know when you have safe water, life becomes very easy. Especially now the children do not get sick, so they are not out of school, and they do not miss classes. We are even going to reduce deaths because children get those infections, and they may end up dying. So we reduce this, increase the retention in schools, and provide clean water in schools.” (KII, ECD).

Role of political governance in WASH

The participants highlighted a lack of government commitment to the provision of WASH commodities, e.g. WaterGuard. This results in reliance on external partners.

“I think unless we have a funding agency or a donor, the government is not forthcoming most of the time. But we can have a donor who can supply the WaterGuards to the schools. But in most cases, we get a short supply, which is not adequate. They supply maybe to the ECDs at intervals. We may stay up to a year without even getting the supply.” (KII, PHO)

“…..we need the county government to take supply of water as a priority, especially to the school and the public health to be involved.” (FGD, Teacher).

Water backpack usage in storage and transport of water

The water backpack was introduced as an innovation geared to aiding school-going pupils in the transport, storage, and dispensing of water. The backpack is a portable product that allows pupils to carry water on their back with straps instead of the conventional jerrycans that can be cumbersome, and the water easily contaminated.

“The way I see that bag, first it's the way of transport, carrying it is so good (easy)……. in the community, there are times we go to ceremonies with these bags, and they are easy to carry. Also, during travel, it is convenient to hang them at the venue, use them, carry them even on the back, and just take them wherever you want.” (KII, Senior CHV)

“… one of the best things I came to learn of the water bags, is it’s one of the best modes whereby it can be utilized anywhere, you can move with it wherever you’re going. Even you can have one at a farm.” (KII, MCA)

The respondents lauded the backpack as being more hygienic, easy to transport, having a larger storage capacity, and easy to clean. They also highlighted the backpack’s durability as a key feature of preference.

“…on the transportation and storage, the backpacks are convenient to the user and even the ones you’ll be dispensing the water to. So, the only shortfall is the, the opening as it's subject to contamination if not well handled. But the package is usually good, especially for the ECDs that we started with. The backpacks are more sanitary than the jerrycans, which you cannot clean properly. They are more hygienic to the users and the community. And it is also comfortable when you’re carrying it because when it is on the back, it’s more stable unlike the jerrycans that you hold using one handle, which are not well balanced. Again, when it comes to cleaning the backpack, it can easily be cleaned once you remove.” (KII, PHO)

“The bags can hold more water compared to the jerrycans. Again, they are convenient because when they are going home they drain the extra water, you fold them, and store in the rooms. They can take less space for storage.” (FGD Teacher)

Backpack use in handwashing

The role of the backpack in strengthening public health hygiene and sanitation through hand washing was emphasized. It was used in most schools as a component of handwashing where it was hung outside classrooms and toilets.

“When they go to the toilet, they know the hygiene. They all know after visiting the toilet you pass by the waterpack and clean your hand. It is known in the schools now.” (KII, ECD)

*“*I can say that washing of hands has become a norm because you see a pupil from the toilet running direct to the (waterpack) point to clean their hands, whether the teacher is in class or not, even if they are late, they doesn't care, they have to clean their hands” (FGD, Teacher)

Evidence of backpack utilization

The respondents referred to the use of backpacks in community social events to ensure access to clean water.

“ …. whenever you have a function somewhere, it can be placed in a strategic position for the purpose of the members of the public. Because at times we have a function where you try to source water points far and wide but a few waterpacks within the community will be of very much benefit.” (KII, MCA)

The backpack helped in reducing pupils’ congestion around water points, offering more points in which pupils can access water.

“….according to our school observations, the water backpacks have helped to reduce the congestion at water points in schools. We put them in classes so there’s no congestion at the water points.” (FGD Teacher)

The backpack was viewed as a beneficial intervention in increasing access to safe drinking water in schools. This is evident in this reflection from the focus groups with teachers.

“This activity is very good because since the water backpacks were provided to schools by you, we have seen a lot of improvements, first of all, our children who have problems getting clean water are now able to get water because these bags are hung in front of every class. So every class has a bag for that purpose and we encourage our pupils to use that water purely for drinking.” (FGD, Teacher)

## Discussion

The Kenya Health Policy objective on elimination of communicable conditions mentions two vital priority policy strategies touching on WASH (1) promotion of good hygiene and sanitation to control water and foodborne diseases; (2) increasing access to improved water safety and sanitations [23]. The findings of this study present perspectives of multisectoral stakeholders in primary school health and WASH. The participant representation was drawn from teachers, school administrators, governance and leadership and community health. The findings are summarized under three domains: contextual, evidence, and facilitation elements, in line with the PARIHS framework [24].

Evidence elements entailed utilization of the water backpack in storage and transport, handwashing leveraged on general backpack utilization. The perspectives of various key players in school health and WASH suggested the applicability of the backpack in strengthening handwashing, storage, and transport of water. Contextual elements featured included WASH strategies in schools and availability of safe water. Key findings showed an association of diarrhea outbreak with unsafe hygiene practices compounded by water scarcity. Similarly, Freeman et al. [16] indicated water-supply improvement, hygiene promotion and water treatment and sanitation interventions resulted in a reduction in diarrhea incidence. There is need to strengthen hygiene education using an integrated approach [7]. Some of the schools had recurrent shortage of safe drinking water due to adversative environmental conditions attributed to drought and climate change. While it takes time, effort, and resources to modify elements of context, a well-developed multidisciplinary facilitation approach involving all key sectors in water access and WASH can drive the change process [25]. increasing the quantity of water available can help improve educational outcomes through the effect of hydration on concentration, attention, and short-term memory [26].

Facilitation refers to empowering the implementation process of an intervention into practice. There was evidence of gaps in synergy between school health and public health system necessitating multisectoral collaboration and social capital capacity building. Social capital can help in the realization of mutually beneficial and accountable cross-fertilization between sectors and agencies [27]. There is need for intersectoral action for health aimed at integrating systematic health concerns in other sectors’ routine policy processes whilst identifying opportunities for promoting the quality of life. The CHVs play an unparalleled role in WASH strategies. They are a vital asset in the bridging of the interface between the community and the health system and are increasingly involved in WASH behavior change promotion [28]. The place of community-based service delivery in achieving health for all can thus not be ignored for impactful interventions that promise a high return on investment [29]. Consequently, the perspectives herein allude to a vital role of the community in WASH strategies. Tsekleves et al. [30] echo this by broaching on the need to leverage on community engagement through co-design and co-production for wider community ownership and acceptance of WASH interventions. The role of key actors such as political governance in WASH programs indicates a lack in commitment in provision of WASH services. To achieve long-term sustainability of WASH strategies and social accountability, there is need for constructive engagement and coordination on service provision. 

## Conclusions

The invaluable importance of collaborative efforts in ensuring safe drinking water for children in schools is at the heart of ensuring sustained child health, growth, and their academic excellence. The national and county governments play an imperative role in ensuring access and continuous supply of safe drinking water in schools. This is fundamental in efforts towards reducing social inequalities of health among school going children and building their social capital. The adoption and of the water backpack requires robust community health systems championed by public health officials and community health workers to ensure sensitization and acceptance in rural communities. The children in these communities are a valuable resource in household uptake and utilization of water since children and women undertake the chores of fetching water daily. Therefore, creating awareness on safe drinking water among school going children will benefit the community. Participatory, collaborative, multisectoral interventions and decision making are crucial, leveraging on creating local ownership, in meeting the water consumption needs of children and communities in water scarce regions.
